# A new method to quantify tau pathologies with ^11^C-PBB3 PET using reference tissue voxels extracted from brain cortical gray matter

**DOI:** 10.1186/s13550-016-0182-y

**Published:** 2016-03-12

**Authors:** Yasuyuki Kimura, Hironobu Endo, Masanori Ichise, Hitoshi Shimada, Chie Seki, Yoko Ikoma, Hitoshi Shinotoh, Makiko Yamada, Makoto Higuchi, Ming-Rong Zhang, Tetsuya Suhara

**Affiliations:** Molecular Imaging Center, National Institute of Radiological Sciences, 4-9-1 Anagawa, Inage-ku, Chiba, Chiba 263-8555 Japan; Division of Neurology, Kobe University Graduate School of Medicine, 7-5-1 Kusunoki-cho, Chuo-ku, Kobe, 650-0017 Japan

**Keywords:** PET quantification, Tau, ^11^C-PBB3, Alzheimer’s disease, Reference tissue

## Abstract

**Background:**

Quantitative in vivo imaging of tau pathologies potentially improves diagnostic accuracy of neurodegenerative tauopathies and would facilitate evaluation of disease-modifying drugs targeting tau lesions in these diseases. Tau pathology can be quantified by reference tissue models without arterial blood sampling when reference tissue devoid of target binding sites is available. The cerebellar cortex has been used as a reference region in analyses of tau positron emission tomography (PET) data in Alzheimer’s disease (AD). However, in a significant subset of tauopathies such as progressive supranuclear palsy and corticobasal degeneration, tau accumulation may occur in diverse brain regions including the cerebellar cortex. This hampers selection of a distinctive reference region lacking binding sites for a tau PET ligand. The purpose of this study was to develop a new method to quantify specific binding of a PET radioligand, ^11^C-PBB3, to tau deposits using reference voxels extracted from cortical gray matter, which have a low likelihood of containing tau accumulations.

**Methods:**

We reanalyzed ^11^C-PBB3 PET data of seven mild AD patients (ADs) and seven elderly healthy control subjects (HCs) acquired in a previous study. As a standard method, parametric images of binding potential (${BP}_{\mathsf{ND}}^{\ast }$) were initially generated using reference tissue manually defined on the cerebellar cortex. We then constructed a frequency histogram of ${BP}_{\mathsf{ND}}^{\ast }$ values in these parametric images and selected cortical gray matter voxels contained in a certain range of the histogram with a low likelihood of having ^11^C-PBB3 binding sites. Finally, these reference voxels were used for generating new ${BP}_{\mathsf{ND}}^{\ast }$ parametric images.

**Results:**

Reference tissue voxels defined by the histogram analysis spread throughout the cortical gray matter of AD and HC brains. The ${BP}_{\mathsf{ND}}^{\ast }$ values determined by our new method correlated very well with those estimated by the standard method (*r*^2^ = 0.94), although the binding estimates by the current method were slightly higher by ~0.14 than those by the standard method.

**Conclusions:**

We developed and validated a new method enabling quantification of tau lesions that can accumulate in the cerebellum and other extensive brain areas. This method may be applicable to all tauopathy subtypes and various tau PET ligands besides ^11^C-PBB3.

**Trial registration:**

The number is UMIN000009052

## Background

Fibrillary tau aggregates are hallmark neuropathologies in diverse neurodegenerative disorders, including Alzheimer’s disease (AD), progressive supranuclear palsy, corticobasal degeneration, and Pick’s disease, collectively referred to as tauopathies. Imaging of tau lesions in living tauopathy patients has been enabled recently, and in vivo quantification of tau pathologies would improve accuracy of diagnosing these disorders and facilitate objective assessments of anti-tau treatments capable of modifying the disease process. Recently, we developed a radioligand, ^11^C-PBB3 (2-((1*E*,3*E*)-4-(6-(^11^C-methylamino)pyridin-3-yl)buta-1,3-dienyl) benzo[*d*]thiazol-6-ol), for tau imaging with positron emission tomography (PET) [1] and established methods for quantification of tau pathologies in the brains of AD patients using ^11^C-PBB3 [2].

With ^11^C-PBB3 PET, AD tau pathology could be accurately quantified by a dual-input graphical analysis with blood data or by an original multilinear reference tissue model (MRTM_O_) without blood data [2]. Noninvasive PET assays without blood sampling are of particular significance for safely conducting scans in demented subjects, highlighting advantages of MRTM_O_. Reference tissue models including MRTM_O_ employ time-activity data in a brain area lacking specific binding components as input functions, and the cerebellum has been utilized as reference tissue in AD since tau does not markedly accumulate in the cerebellar cortex [3]. In other tauopathies, such as progressive supranuclear palsy and corticobasal degeneration, however, tau deposits may be found in extensive cortical and subcortical regions including the cerebellar cortex, cerebellar dentate nucleus, brainstem, thalamus, and basal ganglia [4–6]. This impedes selection of a distinctive brain region devoid of tau ligand binding sites as reference tissue [7, 8]. Thus, a method to extract a reference on a voxel basis needs to be developed in case a single volume with no or minimal binding components cannot be defined for PET quantification.

The purpose of the present study was to develop a new method to quantitatively measure specific binding of ^11^C-PBB3 to tau aggregates using reference voxels extracted from cortical gray matter, which have a low likelihood of containing tau fibrils.

## Methods

^11^C-PBB3 PET data obtained from seven mild AD patients (ADs) and seven elderly healthy control subjects (HCs) in a previous study was reanalyzed (three men/four women for both groups, aged 76 ± 7 years for ADs and 70 ± 6 years for HCs, mean ± standard deviation (SD)) [2]. In brief, mini-mental state examination scores [9] were 19.4 ± 2.4 in ADs and 28.4 ± 2.2 in HCs. All HCs were free of major medical and neuropsychiatric illnesses. All ADs were positive and all HCs were negative for amyloid-β plaques according to amyloid PET with ^11^C-Pittsburgh compound B. This study was approved by the Radiation Drug Safety Committee and the Institutional Review Board of National Institute of Radiological Sciences of Japan. Written informed consent or assent was obtained from all subjects. The study was registered with University Hospital Medical Information Network Clinical Trials Registry (UMIN000009052).

^11^C-PBB3 PET imaging was performed as previously described [2]. After an intravenous injection of ^11^C-PBB3 (injected dose, 399 ± 45 MBq; specific activity, 88 ± 32 GBq/μmol), 70-min dynamic PET scans were conducted using an ECAT Exact HR+ system (developed by CTI, Knoxville, TN, USA, and distributed by Siemens Healthcare, Erlangen, Germany). Arterial blood samples were manually obtained 30 times during the PET scan. Each blood sample was centrifuged, and radioactivity concentrations in the whole blood and plasma were measured. The plasma fractions of the parent and its radiometabolites were determined by high-performance liquid chromatography from six samples. Two-exponential functions were used to interpolate the fraction of the parent and radiometabolites to obtain input functions.

We initially generated parametric images of binding potential (${BP}_{\mathsf{ND}}^{\ast }$) using a standard reference tissue method. A reference region was manually defined on the cerebellar cortex of coregistered magnetic resonance images. Then, parametric images for ${BP}_{\mathsf{ND}}^{\ast }$ were generated for each subject by MRTM_O_ and the cerebellar cortex reference [2]. This approach was based on the assumption that specific radioligand binding was negligible in cerebellar cortical gray matter of the AD brains.

**Fig. 1:**
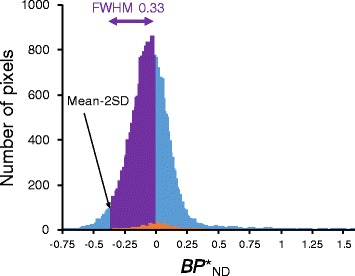
Histogram of ${BP}_{\mathsf{ND}}^{\ast }$ values in a parametric image of a representative HC subject. A frequency histogram was constructed using ${BP}_{\mathsf{ND}}^{\ast }$ values in all voxels (*blue area*). *Purple line* indicates the mean FWHM of a Gaussian distribution fit in HCs (0.33). Voxels with ${BP}_{\mathsf{ND}}^{\ast }$ values above mean −2 SD at a range of the mean FWHM (*purple area*) were selected to generate a reference tissue. A frequency histogram of ${BP}_{\mathsf{ND}}^{\ast }$ values in the manually defined cerebellar cortical regions was indicated in *orange*

We subsequently applied a new method to define reference voxels in cortical gray matter that have a low likelihood of radioligand binding, on the assumption that not all gray matter voxels are affected by tau pathology in most of tauopathies. A frequency histogram of ${BP}_{\mathsf{ND}}^{\ast }$ values was constructed from all voxels of the parametric images generated by the abovementioned standard method (Fig. 1). The histogram of each HC was fitted with a Gaussian distribution function, and mean full width at half maximum (FWHM) of the Gaussian curve in seven HCs was calculated. We defined the reference tissue as a pool of voxels that had ${BP}_{\mathsf{ND}}^{\ast }$ values above mean minus 2 standard deviation (SD) at a range of the mean FWHM (mean ± SD = 0.33 ± 0.03) on the frequency histogram (Fig. 1), followed by more robust selection of voxels in cortical gray matter with the aid of MR segmentation using statistical parametric mapping (SPM12; Wellcome Trust Centre for Neuroimaging, London, UK). The rationale for selecting the mean ${BP}_{\mathsf{ND}}^{\ast }$ range of FWHM was to obtain an adequate total volume of the reference tissue (almost all cortical voxels in HCs), which should provide a very stable reference tissue input function for ${BP}_{\mathsf{ND}}^{\ast }$ estimation. The lowest point of this range was set at the −2 SD limit to threshold out noisy voxels. Then, we defined reference tissue in the cortical gray matter of the AD brains by pooling voxels with ${BP}_{\mathsf{ND}}^{\ast }$ above −2 SD of ADs at a range of the mean FWHM of HCs (=0.33). Finally, a ${BP}_{\mathsf{ND}}^{\ast }$ parametric image was generated for each subject by MRTM_O_ and an individual time-activity curve that were generated by averaging data in these reference voxels. We extracted reference voxels that have a low likelihood of tau binding only from the cortical voxels. In this regard, we could also have included the white matter voxels with a low likelihood of tau binding. However, for ^11^C-PBB3, non-displaceable (nonspecific) binding appears to be different between the gray and white matters [2]. Therefore, we extracted reference voxels only from the cortex in the present study.

In order to compare the two reference tissue definition approaches, we applied the same sets of regions of interest (ROIs) to the parametric images generated by the cerebellar cortical reference tissue and extracted reference tissue methods to obtain mean ${BP}_{\mathsf{ND}}^{\ast }$ values in each ROI as described in the previous paper [2]. For ADs, we generated three to four cerebral cortical ROIs by pooling voxels classified into those with high (>0.3, high), medium (0.15–0.3, middle), low (0–0.15, low), and no (<0, nonbinding) ${BP}_{\mathsf{ND}}^{\ast }$ values on the preliminary parametric images generated by applying coregistered cerebral cortical masks (two AD subjects did not have any significant number of high ${BP}_{\mathsf{ND}}^{\ast }$ voxels, and therefore, three cortical ROIs for these two subjects and four cortical ROIs for the other five AD subjects). These four sets of ROIs had a sample volume of 12 ± 6, 50 ± 27, 103 ± 35, and 188 ± 64 cm^3^, respectively. For HCs, we created one large entire cortical ROI (440 ± 34 cm^3^), since cerebral cortical ${BP}_{\mathsf{ND}}^{\ast }$ was uniformly low.

To investigate difference in the amount of nonspecific binding between the two reference tissues, we measured distribution volumes in these tissues. A dual-input graphical analysis with an arterial input function was used to measure α (a slope of a regression in the graphical analysis, which equals to the weighted sum of total distribution volume of parent and radiometabolites; see Eqs. 1 and 3 in [2]).

## Results

**Fig. 2:**
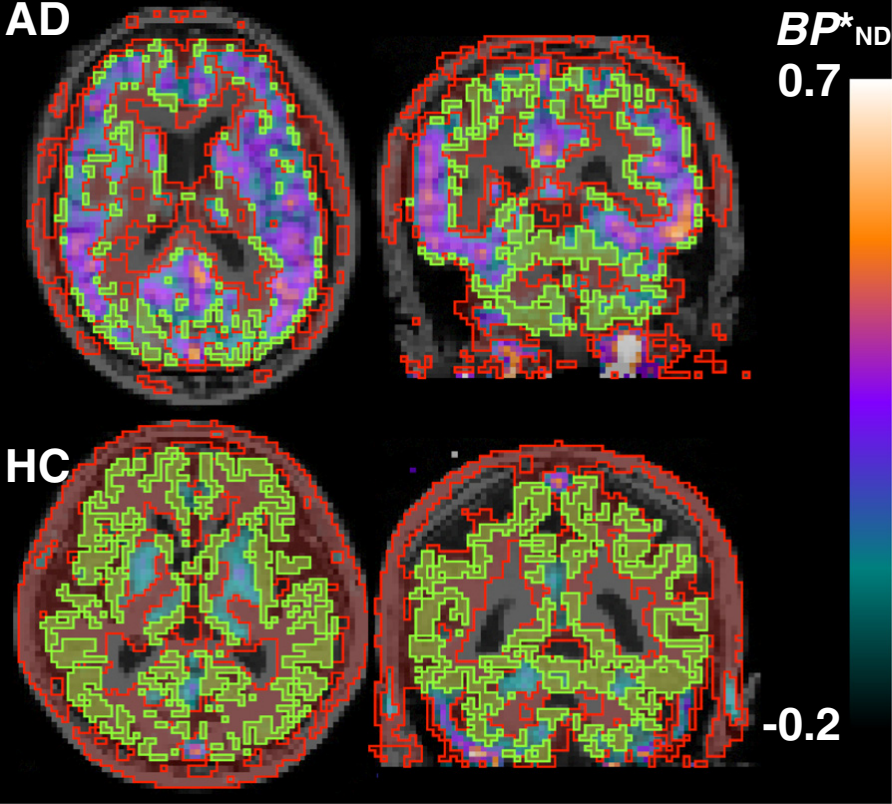
Reference voxels extracted from cortical gray matter of a representative Alzheimer’s disease patient (AD, *top row*) and a healthy control subject (HC, *bottom row*). On transaxial (*left*) and coronal (*right*) ${BP}_{\mathsf{ND}}^{\ast }$ parametric images, areas surrounded by *red lines* indicate voxels with ${BP}_{\mathsf{ND}}^{\ast }$ values above mean −2 SD in this subject for a range of the mean FWHM in HCs (0.33). To obtain reference voxels (*light green area*) extracted from cerebral and cerebellar cortical gray matter, we intersected the *red area* with gray matter voxels segmented on magnetic resonance images

Reference tissue voxels with a low likelihood of noticeable tau deposition selected by our new method broadly spread in cerebral and cerebellar cortical gray matters of the AD and HC brains (Fig. 2). Total volumes of the reference voxels in ADs and HCs were 225 ± 52 and 418 ± 66 cm^3^, respectively.

**Fig. 3:**
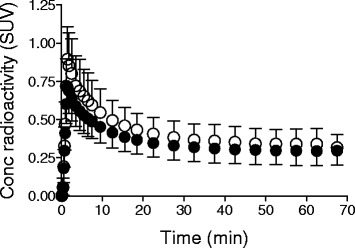
Mean time-activity curves of the new reference tissue extracted from cortical gray matter (*filled circle*) and standard cerebellar cortical reference (*open circle*). Data represent mean ± SD of all subjects

**Fig. 4:**
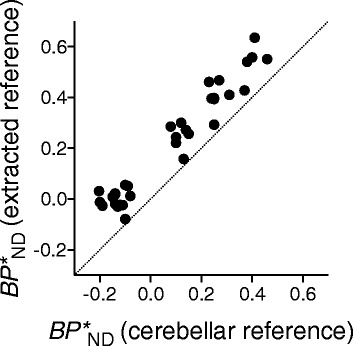
A correlation of ${BP}_{\mathsf{ND}}^{\ast }$ values estimated by MRTM_O_ with the new pooled voxels and standard cerebellar cortical references. A good correlation was observed between parameter estimates by the two methods (*r* ^2^ = 0.94). The *data points* represent ${BP}_{\mathsf{ND}}^{\ast }$ values in each ROI of ADs (3–4 ROIs each) and HCs (one ROI each). *Straight line* indicates line of identity

To validate the applicability of this method to quantification of tau lesions in AD, we compared ${BP}_{\mathsf{ND}}^{\ast }$ values estimated by MRTM_O_ with reference tissues defined by the standard and new methods. The mean time-activity curve in the pooled reference voxels yielded by the new method was slightly lower than that in the cerebellar cortical ROI by the standard method (Fig. 3). The two ${BP}_{\mathsf{ND}}^{\ast }$ values were tightly correlated with each other (*r*^2^ = 0.94, Fig. 4), although the ${BP}_{\mathsf{ND}}^{\ast }$ values calculated by the new method were slightly higher by ~0.14 than those estimated by the standard method using a cerebellar cortical ROI.

**Fig. 5:**
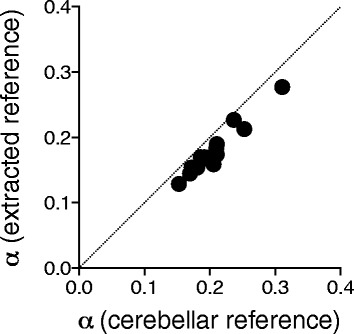
Correlation of α values in two reference regions. α values in the reference voxels extracted from cortical gray matter showed a good correlation with those in the cerebellar cortical ROI (*r* ^2^ = 0.94). *Straight line* indicates line of identity

We also performed a dual-input graphical analysis of PET images in these subjects with arterial input functions to measure α of the reference tissues defined in two ways. The α values in the reference tissue defined by the new method (0.20 ± 0.05 in ADs and 0.16 ± 0.02 in HCs) were slightly lower than those defined by the standard method with the cerebellar cortical ROI (0.22 ± 0.05 in ADs and 0.19 ± 0.02 in HCs), while they were well correlated with each other (*r*^2^ = 0.94, Fig. 5). These results suggest that ${BP}_{\mathsf{ND}}^{\ast }$ values estimated by MRTM_O_ with the new reference voxels are valid, but the non-displaceable distribution of radioligands in these voxels of the HC and AD brains was slightly lower than that in the cerebellar cortex, resulting in slightly higher ${BP}_{\mathsf{ND}}^{\ast }$ values in the target regions.

## Discussion

We documented development of a new method to quantify specific binding of ^11^C-PBB3 to tau pathologies using reference voxels extracted from cortical gray matter. As ${BP}_{\mathsf{ND}}^{\ast }$ values calculated using extracted reference voxels were well correlated with those calculated using cerebellar cortical reference regions in mild ADs and HCs, the new reference method may be used for patients with other tauopathies. Tau accumulation in the cortical gray matter of other tauopathies is often less extensive than that in AD resulting in extraction of large volumes of reference tissue providing reliable input functions for the reference tissue model ${BP}_{\mathsf{ND}}^{\ast }$ estimations.

The ${BP}_{\mathsf{ND}}^{\ast }$ values estimated using the extracted reference voxels were slightly higher than those estimated using cerebellar cortical ROIs. This difference is attributable to the difference in nonspecific binding of the radioligand between the two reference tissues. In addition, radioactivity in cerebellar cortical ROIs can be overestimated by spillover of radioactivity from veins in and adjacent to the cerebellar cortex. In the previous study, we found that non-displaceable distribution volumes in the cerebral cortex were 11–15 % lower than those in the cerebellar cortex of the AD and HC brains [2]. Similarly, we found in this study that α was 11–15 % lower in the extracted reference tissue, which consisted of gray matter from the combined cerebral and cerebellar cortices, than in the cerebellar cortex only reference tissue.

The lower nonspecific binding in the extracted reference voxels may provide the reason why the cerebellar cortical reference tissue constantly gave slightly negative ${BP}_{\mathsf{ND}}^{\ast }$ values in the “nonbinding” cortical gray matter regions without tau accumulation (Fig. 4; −0.14 ± 0.05 in HCs and −0.13 ± 0.03 in ADs). However, the use of the extracted reference voxels resulted in ${BP}_{\mathsf{ND}}^{\ast }$ values in these nonbinding regions close to zero (0.03 ± 0.02 in HCs and −0.03 ± 0.03 in ADs). Thus, the new method appears superior to the standard method in estimating ${BP}_{\mathsf{ND}}^{\ast }$ values in the cerebral cortex, although ${BP}_{\mathsf{ND}}^{\ast }$ may be biased in subcortical areas and white matter, where nonspecific binding may be different from that in the extracted cortical reference tissue.

We also found that α in the extracted reference regions was higher in ADs than in HCs. This finding may stem from low-level contamination of the extracted reference tissue with voxels containing tau lesions in ADs. Theoretically, it is not possible to perfectly discriminate voxels without tau accumulation from those with accumulation in the AD brain by the frequency histogram. Even if the reference tissue is not devoid of specific binding sites, MRTM_O_ calculates a target-to-reference tissue ratio minus 1 at equilibrium, resulting in underestimation of ${BP}_{\mathsf{ND}}^{\ast }$.

To apply the current method to the quantification of tau accumulation in the patients with tauopathies, prior knowledge of HC histograms is required to determine the range of ${BP}_{\mathsf{ND}}^{\ast }$ values to extract reference voxels from the histogram of the patients. The FWHM of the voxel frequency distribution of ${BP}_{\mathsf{ND}}^{\ast }$ values across the brain cortex of the patients was much wider in ADs than that in HCs, because the frequency histogram in the former includes voxels with tau accumulation as opposed to the frequency distribution with all voxels with no tau accumulation in the latter. Therefore, the range of ${BP}_{\mathsf{ND}}^{\ast }$ values for a particular subject cannot be determined using only the FWHM of that subject if the subject is expected to show tau accumulation, because tau accumulations might be underestimated if we used the FWHM ${BP}_{\mathsf{ND}}^{\ast }$ range from that person due to the reference tissue being not devoid of tau binding. However, not all cortical voxels are tau binding positive in tauopathy brains, and this method is intended to extract those voxels with a low likelihood of tau accumulation to be used as the reference tissue. For this reason, the current method requires the mean FWHM of HCs as the lower range of ${BP}_{\mathsf{ND}}^{\ast }$ with an assumption that those voxels in this range should have a low likelihood of tau accumulation.

Although we used manually defined reference regions on the cerebellar cortex, the initial parametric images can be generated using automatically segmented cerebellar cortices. The final target ${BP}_{\mathsf{ND}}^{\ast }$ values will be the same for the two reference region selection methods. It is because that the shape of histograms will be the same regardless of the choice of the methods to define the cerebellar cortical reference region for the same subject. If the cerebellar cortical reference ROI includes tau binding voxels, the origin of the histogram (the point of ${BP}_{\mathsf{ND}}^{\ast }$ = 0) on the horizontal ${BP}_{\mathsf{ND}}^{\ast }$ bin axis will be shifted to the right (i.e., the histogram will be shifted to the left), but the histogram will have the same ${BP}_{\mathsf{ND}}^{\ast }$ voxel distribution shape as in the case where no tau binding voxels are included in the cerebellar cortical ROI. That is, ${BP}_{\mathsf{ND}}^{\ast }$ in some target regions can have negative values if this is the final step. However, this step is a preliminary one in the current method. In the current method, we extract reference voxels that fall under the lower end of the histogram range, which sets the mean ${BP}_{\mathsf{ND}}^{\ast }$ in this range to be zero in the regenerated ${BP}_{\mathsf{ND}}^{\ast }$ parametric images.

Of note is that the commonly used standardized uptake value ratio (SUVR) method also requires identification of reference tissue regions devoid of tau binding to be used as the denominator for calculating the target-to-reference tissue SUV ratios. Therefore, the lack of distinctively identifiable reference regions in tauopathies presents the same problem as for the ${BP}_{\mathsf{ND}}^{\ast }$ method. In this sense, the current method of extracting voxels with a low likelihood of tau binding might also be applicable to the SUVR method by generating voxel SUV frequency histograms in both healthy controls and tauopathy patients.

## Conclusions

In conclusion, we demonstrated the validity of a new method to assess tau pathologies with ^11^C-PBB3 using cortical gray matter reference tissue, by the use of PET data in ADs and HCs. This method may be applicable to patients with non-AD tauopathies with diverse tau distribution.

### Ethics approval and consent to participate

This study was approved by the Radiation Drug Safety Committee and the Institutional Review Board of National Institute of Radiological Sciences of Japan (#12-018). All the procedures performed in the studies involving human participants were in accordance with the ethical standards of the institutional and/or national research committee and with the 1964 Helsinki declaration and its later amendments or comparable ethical standards. Written informed consent or assent was obtained from all the subjects.

### Consent for publication

Not applicable.

## Abbreviations

$$ {BP}_{\mathsf{ND}}^{\ast } $$: binding potential
^11^C-PBB3: 2-((1*E*,3*E*)-4-(6-(^11^C-methylamino)pyridin-3-yl)buta-1,3-dienyl) benzo[*d*]thiazol-6-ol
AD: Alzheimer’s disease
FWHM: full width at half maximum
HC: healthy control
MRTM_O_: original multilinear reference tissue model
PET: positron emission tomography
SD: standard deviation
